# Dopamine neurons in the ventral periaqueductal gray modulate isoflurane anesthesia in rats

**DOI:** 10.1111/cns.13447

**Published:** 2020-09-02

**Authors:** Chengxi Liu, Xiao Zhou, Qiuyu Zhu, Bao Fu, Song Cao, Yu Zhang, Lin Zhang, Yi Zhang, Tian Yu

**Affiliations:** ^1^ Guizhou Key Laboratory of Anesthesia and Organ Protection Zunyi Medical University Zunyi China; ^2^ Guizhou Key Laboratory of Brain Science Zunyi Medical University Zunyi China; ^3^ Department of Critical Care Medicine Affiliated Hospital of Zunyi Medical University Zunyi China; ^4^ Department of Anesthesiology The Second Affiliated Hospital of Zunyi Medical University Zunyi China

**Keywords:** calcium fiber photometry recording, dopamine, GABA_A_ receptor, isoflurane, righting reflex, ventral periaqueductal gray

## Abstract

**Aims:**

General anesthesia has been applied in surgery for more than 170 years, and there is little doubt that GABA_A_ receptors have an important role as anesthetic molecular targets, but its neural mechanisms remain unclear. Increasing researchers have shown that dopaminergic pathways in the brain are crucial for sleep and wake. General anesthesia‐induced unconsciousness and natural sleep share some neural correlates. However, the role of GABA_A_ receptors in ventral periaqueductal gray (vPAG) dopamine (DA) neurons in the isoflurane‐induced unconsciousness has yet to be identified.

**Methods:**

In the present study, we used calcium fiber photometry recording to explore that the activity of ventral periaqueductal gray (vPAG) neurons. Then, rats were unilaterally microinjected with 6‐hydroxydopamine into the vPAG area to determine the role of vPAG‐DA neurons in isoflurane‐induced‐anesthesia. Furthermore, thirty SD rats were divided into three groups: a GABA_A_R agonist‐muscimol group, a GABA_A_R antagonist‐gabazine group, and a control group. Finally, whole‐cell patch clamp was used to examine the effects of isoflurane and GABA_A_ receptor agonist/antagonist on vPAG‐DA neurons.

**Results:**

The vPAG neurons were markedly inhibited during isoflurane anesthesia induction and that these neurons were activated during emergence from isoflurane anesthesia. Lesion to the vPAG‐DA neurons shortened the induction time and prolonged the emergence time while increasing δ power in isoflurane anesthesia. Intracerebral injection of the GABA_A_ receptor agonist (muscimol) into the vPAG accelerated the induction of anesthesia and delayed recovery from isoflurane anesthesia, with a decrease of δ power and an augment of β power. Injection of GABA_A_ receptor antagonist gabazine generated the opposite effects. Isoflurane enhanced GABAergic transmission, and GABA_A_ receptor agonist partly increased isoflurane‐induced inhibition of vPAG‐DA neurons, while GABA_A_ receptor antagonist evidently attenuated GABAergic transmission.

**Conclusion:**

Our results suggest that vPAG‐DA neurons are involved in isoflurane anesthesia through activation of the GABA_A_ receptor.

## Graphical abstract

General anesthesia has been widely applied in surgery for more than 170 years. However, its underlying mechanisms are still a mystery. It was reported that dopaminergic pathways in the brain are crucial for the emergence from general anesthesia. We previously also found that reductions of ventral periaqueductal gray (vPAG) dopaminergic neurons numbers shortened the induction time and prolonged the emergence time from propofol anesthesia. However, the role of GABA_A_ receptor in ventral periaqueductal gray (vPAG) dopamine (DA) neurons in the isoflurane‐induced unconsciousness has yet to be identified. Here, using real time in vivo fiber photometry, we found that neuronal activity in the vPAG was markedly disinhibited during the recovery from isoflurane anesthesia. Subsequently, microinjection of 6‐hydroxydopamine (6‐OHDA) into the vPAG to selectively kill DA neurons shortened the induction time and prolonged the emergence time, with an increase of δ‐band power in cortical electroencephalogram (EEG). Then, microinjection of GABA_A_ receptor selective agonist (muscimol) into the vPAG notably accelerated the induction of anesthesia and delayed recovery from isoflurane anesthesia, with a decrease of δ power and an augment of β power. Microinjection of GABA_A_ receptor antagonist (gabazine) generated the opposite effects. Finally, whole‐cell patch clamp was applied to further explore the cellular mechanism underlying modulation of GABA_A_ receptor on vPAG‐DA neurons under isoflurane anesthesia. Our data demonstrated that the frequency of spontaneous inhibitory postsynaptic current (sIPSC) of vPAG‐DA neurons was enhanced by isoflurane. GABA_A_ receptor agonist enhanced the effect of isoflurane on sIPSC, and its actions were eliminated by application of gabazine. Collectively, these results indicate that isoflurane activates GABA_A_ receptor to affect GABA neuron and vPAG‐DA neurons. Then, isoflurane directly suppressed the activity of wake‐promoting vPAG‐DA neurons to induce anesthesia or activate GABA neuron to indirectly inhibit vPAG‐DA neurons causing the reduction of DA. Our results suggest that vPAG‐DA neurons are involved in isoflurane anesthesia through activation of the GABA_A_ receptor.

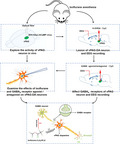

## 1 INTRODUCTION

General anesthesia is a reversible state characterized by loss of consciousness and has been widely applied in surgery for more than 170 years. However, its underlying mechanisms are still a mystery. Recent data have indicated that the transitions into both sleep and general anesthesia share some neuronal mechanisms.^1^, ^2^, ^3^ Importantly, dopaminergic pathways in the brain are vital for regulation of sleep and wake.^4^, ^5^, ^6^ In the clinic, methylphenidate and amphetamine, which elevate extracellular dopamine (DA) concentrations, are commonly applied for treating attention‐deficit hyperactivity disorder and sleep disorders such as narcolepsy.^7^, ^8^, ^9^ Additionally, systemic administration of D1 receptor agonist^10^ or methylphenidate^11^ strongly quickened the reanimation from isoflurane anesthesia in rats.^12^ It is interesting to note that optogenetic activation of DA neurons in the VTA induces reanimation from isoflurane anesthesia to an awake state, which hinted that DA neurons in distinct regions play different roles depending on the general anesthetic agent used.^13^ These studies indicated that the dopaminergic system plays an important role in both general anesthesia and the sleep‐wake process, although it remains unclear whether dopaminergic circuits in the brain regulate wakefulness.

The ventral periaqueductal gray (vPAG) is a key neuronal region that mediates a variety of behaviors such as pain, fear, anxiety, and wakefulness.^4^, ^14^ More than 10 years ago, researchers found that lesions in DA neurons of the ventral periaqueductal gray area (vPAG) promote sleep and that the vPAG has diverse interconnections with arousal‐ and sleep‐relevant regions.^15^ We previously found that reductions of vPAG‐DA neurons numbers shortened the induction time and prolonged the emergence time from propofol anesthesia,^16^ contradicting previous findings that the dopaminergic system only affected recovery processes in general anesthesia.^10^, ^11^, ^16^ Moreover, lesions in DA neurons of the VTA significantly delayed reanimation from propofol without affecting the processes of isoflurane or ketamine anesthesia.^17^ However, Taylor et al discovered that photostimulation of DA neurons in the VTA led to transition from isoflurane‐induced anesthesia to an awake state, which hinted that DA neurons in distinct regions play different roles depending on the general anesthetic agent used.^13^ Therefore, we further studied whether DA neurons in the vPAG are involved in regulating isoflurane anesthesia.

In the present study, calcium fiber photometry recordings were used to examine the neural activities of vPAG in the process of isoflurane‐induced anesthesia. Then, 6‐hydroxydopamine (OHDA) was used to selectively kill DA neurons in Sprague Dawley rats and ascertain the functional type of vPAG seen during isoflurane anesthesia. Subsequently, the GABA_A_ receptor in the vPAG was modulated using a pharmacological approach to explore the mechanisms by which isoflurane induces general anesthesia, and the impact of isoflurane on GABAergic transmission in the vPAG was determined using whole‐cell patch clamp recording.

## 2 MATERIALS AND METHODS

### 2.1 Animals

This study was approved by the Animal Care and Use Committees of Zunyi Medical University. Animal studies were performed in accordance with the Guide for the Care and Use of Laboratory Animals in China (No. 14924, 2001). Male Sprague Dawley rats (weight: 260‐330 g, age: 12‐16 weeks) were provided by the Animal Center of the Third Military Medical University (Chongqing, China). Rats were housed in standard chambers within an SPF laboratory animal room (12/12‐hour light/dark cycle; 23 ± 2°C; relative humidity: 55% ± 2%). They were given free access to water and food. To abate the confounding effects of circadian timing on the experimental results, all behavioral tests and EEG experiments were performed between 6:00 pm and 12:00 pm.

### 2.2 Drugs

Isoflurane was purchased from RWD Life Science (Shenzhen, China). Pentobarbital and lidocaine were purchased from Chaohui Pharmaceutical. 6‐OHDA, AP‐5, DNQX, strychnine, muscimol, and gabazine were purchased from Sigma‐Aldrich. CY3 Donkey anti‐rabbit IgG, Mouse and Rabbit Specific HRP/DAB (ABC), Detection IHC kit, and rabbit antityrosine hydroxylase are products of Abcam Corp. 6‐OHDA hydrochloride (Sigma) was dissolved in 0.9% saline containing 0.2% ascorbic acid to avoid decomposition.

### 2.3 Calcium fiber photometry recordings

**Figure 1:**
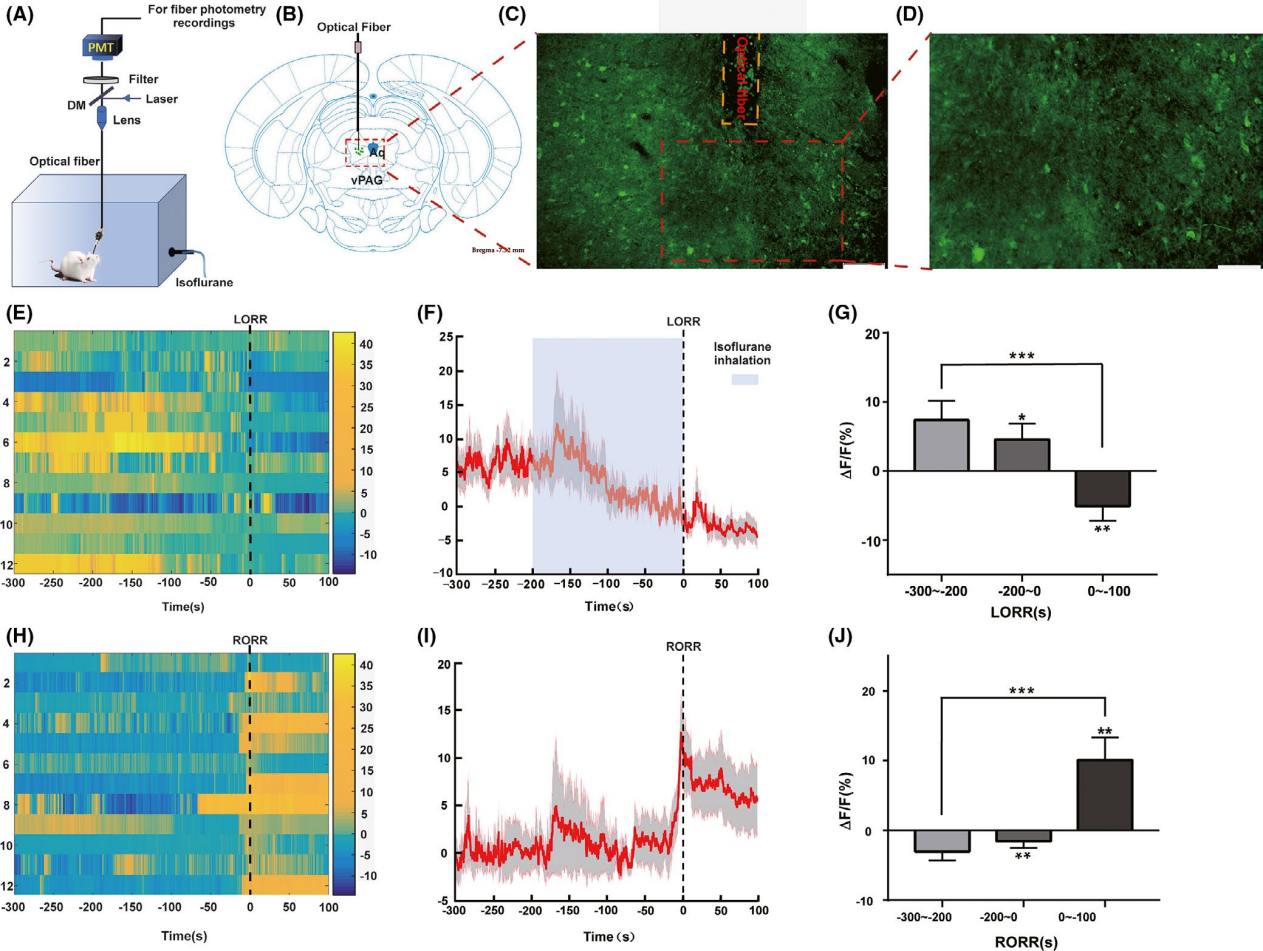
Phase‐dependent calcium alterations in ventral periaqueductal gray (vPAG) neurons during isoflurane anesthesia. A, Diagram of experimental setup for in vivo calcium signal recordings. B, Schematic of the AAV‐hSyn‐GcaMP virus’ site, modified from the Rat Brain Atlas. Aq = Aqueduct. C, Histological immunohistochemical photograph showing the AAV‐hSyn‐GcaMP virus vector and fiber injecting sites in the vPAG. (scale bar = 200μm). D, Higher magnification photograph of (C) (scale bar = 100μm). E, Fluorescence calcium signals aligned to isoflurane‐induced loss of righting reflex (LORR). F, Mean (red trace) ± SEM (gray shading) indicating the average calcium transients during isoflurane‐induced LORR (n = 12). G, The fluorescence calcium signals sharply decreased after infusion of isoflurane. H, Fluorescence calcium signals corresponded to isoflurane‐induced recovery of righting reflex (RORR). I, Mean (red trace) ± SEM (gray shading) showing the transients of average calcium signals during isoflurane‐induced RORR (n = 12). J, The fluorescence calcium signals sharply increased during transition from isoflurane‐induced anesthesia to arousal (*P < .05; **P < .01; ***P < .0001)

Pentobarbital (50 mg/kg) was injected intraperitoneally to anesthetize the rats (n = 12); prior to surgery, lidocaine (2%) was injected subcutaneously to induce local anesthesia. Subsequently, the rats were gently placed on a stereotaxic frame (RWD Life Science, Shenzhen, China), and the AVV‐hSyn‐GCaMP virus expression vector was injected unilaterally into the vPAG area (250 nL/side; speed: 50 nL/min) (anterior‐posterior [AP]: −7.3 mm, medial‐lateral [ML]: +0.6 mm, and dorsal‐ventral [DV]: −5.4 mm, Figure 1B) through a glass micropipette using a microsyringe pump. The injection was guided by stereotactic coordinates from the rat brain atlas (Paxinos & Watson, 2007). Next, an optical fiber (OD: 200 μm, numerical aperture: 0.37; Newton Inc) was implanted and fixed to the skull using a skull‐penetrating screw and dental cement. After 28 days, the animals received isoflurane anesthesia for further testing.

Using a multichannel fiber photometry system (ThinkerTech Nanjing Bioscience Nanjing) equipped with a 480‐nm excitation LED (3W, CREE) and a dichroic mirror (DCC3420M; Thorlabs), the fluorescence signals of the GCaMP were recorded using multifunction data acquisition software (Thinker Tech Nanjing Bioscience Inc), simultaneously filtered at 40 Hz, and digitalized at 500 Hz. An optical fiber (Newton Inc) integrated with an optical diverter (Doric Lenses) was used to transmit the light between the fiber photometry system and the implanted optical fiber. One month later, the rats were used to record changes in GCaMP signals.

Before anesthesia, 100‐second recording was made. Next, the rats were anesthetized using 1.4% isoflurane; the moment of loss of righting reflex (LORR) and recovery of righting reflex (RORR) were marked, and the recording was stopped 10 minutes after RORR. Isoflurane anesthesia between LORR and RORR was maintained for 25 minutes to ensure that the isoflurane concentration had equilibrated in the brain. Eight rats were subjected to the test two times each, with a 4‐day rest between experiments. All brains were sectioned to verify viral fluorescence expression, and optical fiber implantation after the experiment was completed. Fiber photometry data were analyzed using MATLAB 2016a (MathWorks). The values of fluorescence change (_Δ_ *F*/*F*) were calculated using the following formula: (*F* ‐ *F*_0_)/*F*_0_, where *F* is the test fluorescence signal and *F*_0_ is the mean and standard deviation of the basal signal.

### 2.4 Lesions in the vPAG

For selective depletion of DA Neurons in the vPAG, rats were unilaterally injected with 1 μL of 6‐OHDA (4 μg/μL) into the vPAG area using aseptic technique. The rats in the 6‐OHDA‐lesioned group (n = 10) were microinjected using a glass micropipette and a microsyringe pump (KD Scientific). The neurotoxin 6‐OHDA is commonly used to selectively destroy dopaminergic neurons and is therefore often used to establish Parkinson's disease models.^18^ The rats in the sham group (n = 10) were injected with 1 μL saline into the vPAG using the same procedure. All rats were allowed two weeks to recover before subsequent experiments.

Rats were anesthetized in an anesthesia chamber filled with 1.4% isoflurane and 100% oxygen at a flow rate of 1 L/min after the chamber had equalized for 10 minutes. The LORR time was then recorded. Subsequently, the rats were anesthetized for 25 minutes, with their EEG signals being recorded for 10 minutes before the end of anesthesia (Figure 3A‐C). Immediately after anesthesia, the rats were quickly and gently removed from the anesthesia chamber and placed in the supine position in another chamber to record RORR time.

### 2.5 Behavioral testing

**Figure 2:**
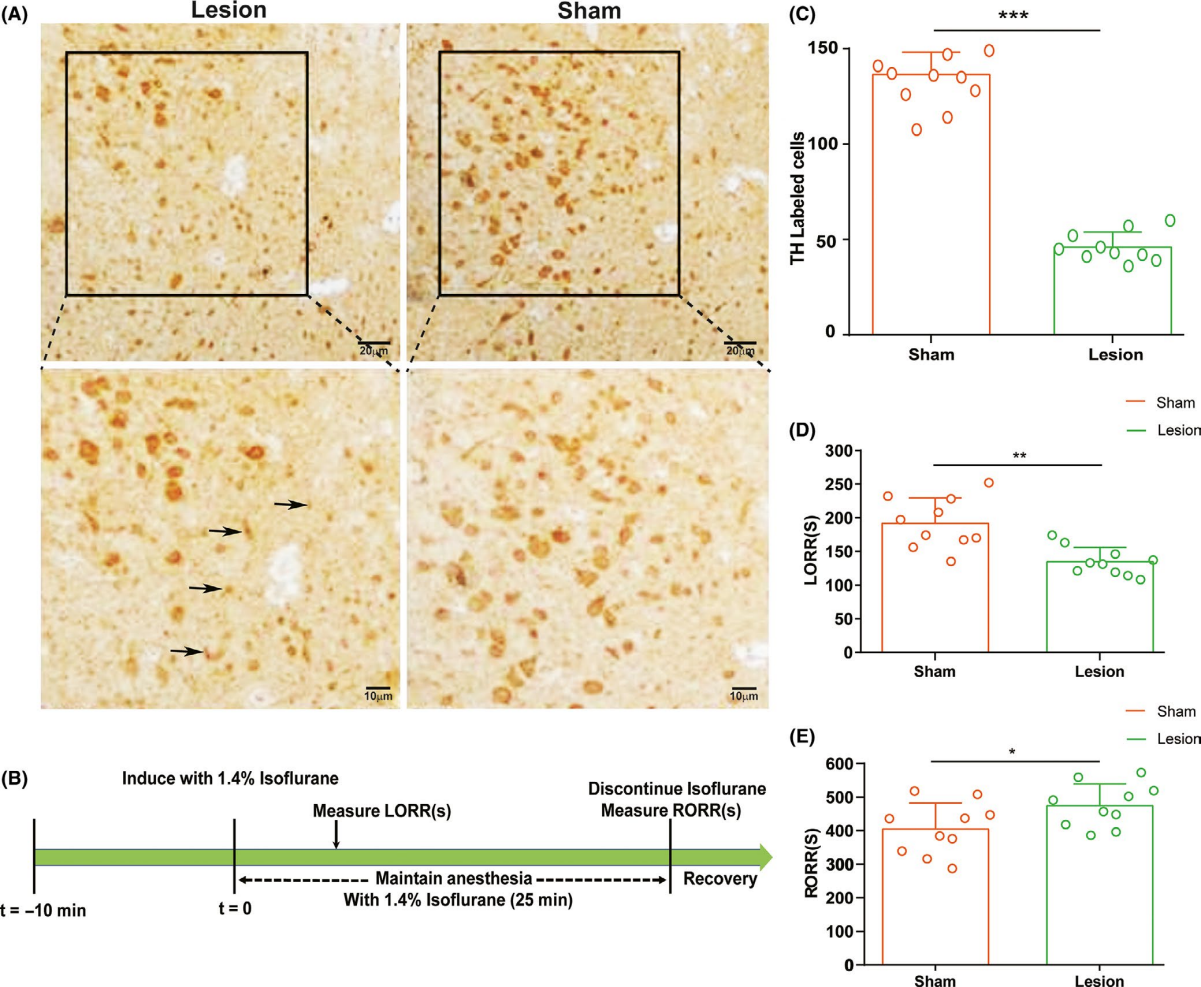
Effect of unilateral DA neuron lesion in the ventral periaqueductal gray (vPAG) on LORR and RORR time of isoflurane anesthesia. A, Representative immunohistochemical image showing unilateral site of lesion group and sham group in the vPAG. The lesion group animals were selectively depleted of DA Neurons in the vPAG with unilaterally injection of 1 μL of 6‐OHDA. Black arrowhead denotes the dead cells. B, Timeline for quantifying induction and recovery time with isoflurane. C, Quantitative analysis of the number of tyrosine hydroxylase‐positive neurons on the lesion and normal sides. D, Induction time and E, recovery time in the lesion and sham groups (n = 10 per group; mean ± SD; *P < .05; **P < .01)

LORR and RORR time in rats is considered a standardized index of the general anesthesia induction and emergence times, respectively. Usually, the anesthesia induction time is regarded as the time to LORR in rats. For this reason, the rats were put into an anesthesia chamber that had been allowed to equalize for 10 minutes. Subsequently, 1.4% isoflurane with oxygen at 1 L/min was delivered into the anesthesia chamber for 25 minutes (Figure 2B). The rats were then removed from the chamber and allowed to emerge from anesthesia. The period from the start of isoflurane treatment to LORR was deemed the LORR time, while the duration from the end of isoflurane infusion to RORR was defined as the RORR time.

For the microinjection experiment, 30 rats were randomly divided into three groups: a GABA_A_R agonist‐muscimol group, a GABA_A_R antagonist‐gabazine group, and a saline group. As described above, a microinjection catheter with two guide cannulas (OD: 0.48 mm × ID: 0.34 mm; length: 5.6 mm) was implanted into the bilateral vPAG. Two additional electrodes were then implanted over the bilateral prefrontal cortex to reduce experimental rats. Finally, the catheters and electrodes were fixed onto the skull with two screws and dental acrylic. One week later, the animals received isoflurane anesthesia as described above to quantify their induction and emergence times. Before LORR time quantification, the rats in each group were microinjected with either saline (1 μL), muscimol (1 μL; 0.5 μg/μL), or gabazine (1 μL; 0.5 μg/μL) into the vPAG via a microsyringe pump (Legato® 130; KD Scientific) at a speed of 0.2 μL/min; *t* = −5 minutes). Subsequently, 1.4% isoflurane with 100% oxygen was continuously delivered into the anesthesia chamber at 1 L/min via an isoflurane vaporizer (Ohmeda). In the recovery time assessment, 1.4% isoflurane was maintained for another 25 minutes to reach steady‐state general anesthesia (Figure 5A).

### 2.6 EEG recording and Spectral analyses

EEGs were recorded at least 5 days after the behavioral test to allow recovery from anesthesia. The CED Power1401‐3 device (Cambridge Electronic Design) and the Spike 2 software package (Cambridge Electronic Design) were used to acquire EEG signals. EEG signals were then amplified using AM Systems 3000 amplifiers (AM Systems Inc). The range of signals was filtered between 0.1 and 300 Hz. After induction, the rat's EEG signals were collected, and anesthesia was maintained for 30 minutes. Muscimol or gabazine was injected 15 minutes before the end of anesthesia. EEG was recorded continuously until the rat recovered from isoflurane anesthesia. Power spectrum analysis was conducted on data recorded 600 seconds before and after the microinjections. Relative powers in the different frequency bands were computed by averaging the signal power across the frequency range of each band (δ: 1‐4 Hz, θ: 4‐8 Hz, α: 8‐12 Hz, β: 12‐25 Hz, and γ: 25‐60 Hz) and then dividing by the total power from 1‐60 Hz. Spectrograms were constructed using multitaper methods implemented using the Chronux toolbox in MATLAB 2016a (MathWorks, Cambridge, UK).

### 2.7 Histological localization of cannula position and immunohistochemistry

Pentobarbital (55 mg/kg) was injected intraperitoneally to anesthetize the rats, and lidocaine (2%) was injected subcutaneously to induce local anesthesia. After deep anesthesia, the rats were transcardially infused with 300 mL of 0.01‐M PBS, followed by 250 mL 4% PFA. Their brains were removed and fixed in 4% PFA overnight at 4°C. The brains were later transferred to 30% sucrose at 4°C until they sank. The brains of the microinjection groups were coronally sectioned into 30‐μm slices in a cryostat (CM1950; Leica) to validate the microinjection sites according to the rat brain atlas (Paxinos & Watson, 2007). For the lesion experiment, the brains were sectioned into slices, as described previously. The dopaminergic neurons of the vPAG were stained immunohistochemically using antityrosine hydroxylase primary antibody. The number of neuronal lesions in the vPAG area was then calculated in a blinded manner by comparing positively immunostained neurons in Image J. Tyrosine hydroxylase‐positive neurons were counted in a 0.5 mm × 0.5 mm box. Cell counting was performed on three adjacent sections (separated by 90 μm) of the brain, and the average counting per section was used to represent the data.

### 2.8 Whole‑cell patch clamp recording

#### 2.8.1 Brain slice preparation

Experiments were conducted on brain slices isolated from young male Sprague Dawley rats (11‐16 days old) under deep isoflurane anesthesia. The rat brains were gently removed and transversely sliced at a thickness of 300 µm using a vibrational microslicer (HM650V; Thermo Fisher Scientific). The brain slices including the vPAG were placed in an incubating solution containing 124 mmol/L NaCl, 3 mmol/L KCl, 1.5 mmol/L KH_2_PO_4_, 24 mmol/L NaHCO_3_, 2 mmol/L CaCl_2_, 1.3 mmol/L MgSO_4_, and 10 mmol/L glucose. The solution was saturated using 95% O_2_ and 5% CO_2_ at a constant temperature (32°C) for 30 minutes. It was then restored to room temperature (22‐25°C) for at least 30 minutes.

#### 2.8.2 Electrophysiology

Whole‐cell patch clamp recordings were applied to record the spontaneous inhibitory postsynaptic currents (sIPSC) of the vPAG neurons. Brain slices were constantly perfused with artificial cerebrospinal fluid (ACSF) saturated with 95% O_2_ and 5% CO_2_ at room temperature. The vPAG neurons were visually identified using a BX51WI microscope (Olympus) equipped with infrared‐differential interference contrast optics, as described previously (18). A patch micropipette (4‐6 MΩ) was pulled from glass capillaries (Sutter) and filled with an internal solution containing 10 mmol/L N‐2‐hydroxyethylpiperazine‐N, −2‐ethanesulfonic acid, 10 mmol/L KCl, 130 mmol/L K‐gluconate, 2 mmol/L MgCl_2_, 2 mmol/L ATP‐Mg, and 0.2 mmol/L GTP‐tris (hydroxy‐methyl) aminomethane (pH, 7.2; osmotic pressure, 280 mOsm).

#### 2.8.3 Whole‐cell voltage‐clamp recordings

To isolate GABAergic spontaneous inhibitory postsynaptic currents, 1 μmol/L strychnine (a selective glycine receptor antagonist), 50 µmol/L AP‐5 (an AMPA receptor antagonist), and 20 µmol/L DNQX (NMDA receptors antagonist) were diluted in ACSF. The membrane potential was maintained at −70 mV. The recording intracellular solution contained 135 mmol/L CsF, 2 mmol/L MgCl_2_, 5 mmol/L KCl, 10 mmol/L Hepes, 0.2 mmol/L GTP, and 2 mmol/L Mg‐ATP; it had a pH of 7.3 and an osmotic pressure of 300‐305 mOsm. All data were acquired using a HEKA EPC10 amplifier (HEKA Instruments, Inc), as previously described. To record currents, the basal noise values were controlled under 10 pA.

#### 2.8.4 Application of drugs

In the present study, isoflurane was applied with a carrier gas (95% O_2_‐5% CO_2_) using calibrated commercial vaporizer. The effective concentration of 1.4% isoflurane dissolved in ACSF is 0.50 mmol/L.^18^ Other drugs dissolved in ACSF were perfused using a BPS‐4 perfusion system (ALA, USA). We started to record the current after 10 minutes of drug application, and the recording time was 5 minutes.

#### 2.8.5 Cell immunofluorescence

To identify the DA neurons, we labeled the recorded neurons using 2% lucifer yellow (green fluorescence in Figure 6B). At the end of recording, the slices were moved to a container, fixed with 4% paraformaldehyde, and incubated in PBS/0.3% Triton X‐100 for 20 minutes. They were then placed in 5% donkey serum in PBS/0.1% Triton X‐100 for 1 hour, then in primary antibody (1:500; rabbit antityrosine hydroxylase; Abcam, ab112) diluted in PBS/0.3% Triton X‐100 containing 5% donkey serum overnight at 4°C. The slices were then washed three times (5 minutes each) in PBS/0.3% Triton X‐100 and incubated in secondary antibody (1:1000; goat anti‐rabbit IgG H&L; Abcam; ab102294) diluted in PBS/0.3% Triton X‐100 for 1 hour at room temperature. An immunofluorescence microscope (Olympus BX63) was used to detect immunofluorescent staining. We strictly selected data in which tyrosine hydroxylase‐positive neurons merged with lucifer yellow for analysis.

### 2.9 Data analyses

All statistical analyses were performed by the GraphPad Prism software package, version 6.0 (GraphPad Software Inc). All data were subjected to tests for normality. The differences in cell count, LORR and RORR time, and EEG power were also detected using unpaired Student's *t* test between the lesion and sham groups. Furthermore, paired Student's *t* tests were used to analyze differences in calcium signals between the pre‐ and postevents, as well as the change in EEG between the premicroinjection and postmicroinjection. Moreover, one‐way ANOVAs were used to compare LORR and RORR times for application of muscimol, gabazine, or saline in isoflurane anesthesia. Additionally, sIPSC were analyzed using the Mini Analysis Program (Synaptosoft Inc). The amplitude and frequency of sIPSC were normalized to their mean value during the control period. Differences in current frequency, amplitude, and decay time were tested using Student's paired two‐tailed *t* test. Data are presented as mean ± SD or mean ± SEM. In all cases, *P*‐values < .05 were considered significant.

## 3 RESULTS

### 3.1 Population‐wide activity of vPAG neurons sharply increased during the reanimation period of isoflurane anesthesia

To examine the real‐time activity of the vPAG during isoflurane anesthesia, GCaMP was expressed in the vPAG neurons; this is a calcium indicator used to image neuronal activity. To this end, the AAV‐hSyn‐GcaMP virus was injected into the vPAG (n = 12). Using fiber photometry technology, we implanted an optical fiber above the vPAG to collect and record the real‐time fluorescent emission from GcaMP during isoflurane anesthesia (Figure 1A,C,D).

During isoflurane anesthesia induction, we analyzed calcium signals in three sections: baseline (wake: −300 to −200 seconds), induction period, and anesthesia period. The 1.4% isoflurane concentration was sufficient to induce LORR over 200 seconds; we defined this as the induction period: from isoflurane infusion to LORR. The anesthesia period was defined as the period from LORR to 100 seconds after LORR. As shown in Figure 1E,F, there was transient growth in the calcium signal after isoflurane infusion; this may have been caused by the rat's struggle and evasion during isoflurane infusion. With the degree of anesthesia increasing, the calcium signal gradually declined during the induction period (baseline: 6.118% ± 2.654% vs. pre‐LORR: 4.544% ± 2.276%; mean ± SEM, *P* = .046 by paired *t* test). Additionally, a gentle reduction in calcium signal occurred from the induction period to the anesthesia period (Figure 1G; pre ‐LORR: 4.544% ± 2.276% vs. post‐LORR: −5.061% ± 2.174%; mean ± SEM, *P* = .006 by paired *t* test), suggesting that the vPAG neurons were inhibited during the inhalation anesthesia phase. The same three sections were analyzed during the emergence period. From cessation of isoflurane anesthesia to arousal, calcium signals were higher than at baseline (−4.154% ± 1.223% vs. pre‐RORR: −1.667% ± 1.152%; mean ± SEM, *P* = .008 by paired t test, Figure 1J). A sharp rising of neuronal activity then occurred in the vPAG concurrent with the moment of RORR (pre‐RORR: −1.667% ± 1.152% vs. post‐RORR: 11.962% ± 3.565%; mean ± SEM, *P* = .002 by paired t test; Figure 1H,I,J). Generally, our results indicated that vPAG neurons are suppressed during isoflurane‐induced anesthesia and activated during the emergence process, hinting that vPAG neurons may be involved in the process of isoflurane anesthesia.

### 3.2 DA neurons in vPAG affected the induction time and the emergence time companied cortical spectral changes

**Figure 3:**
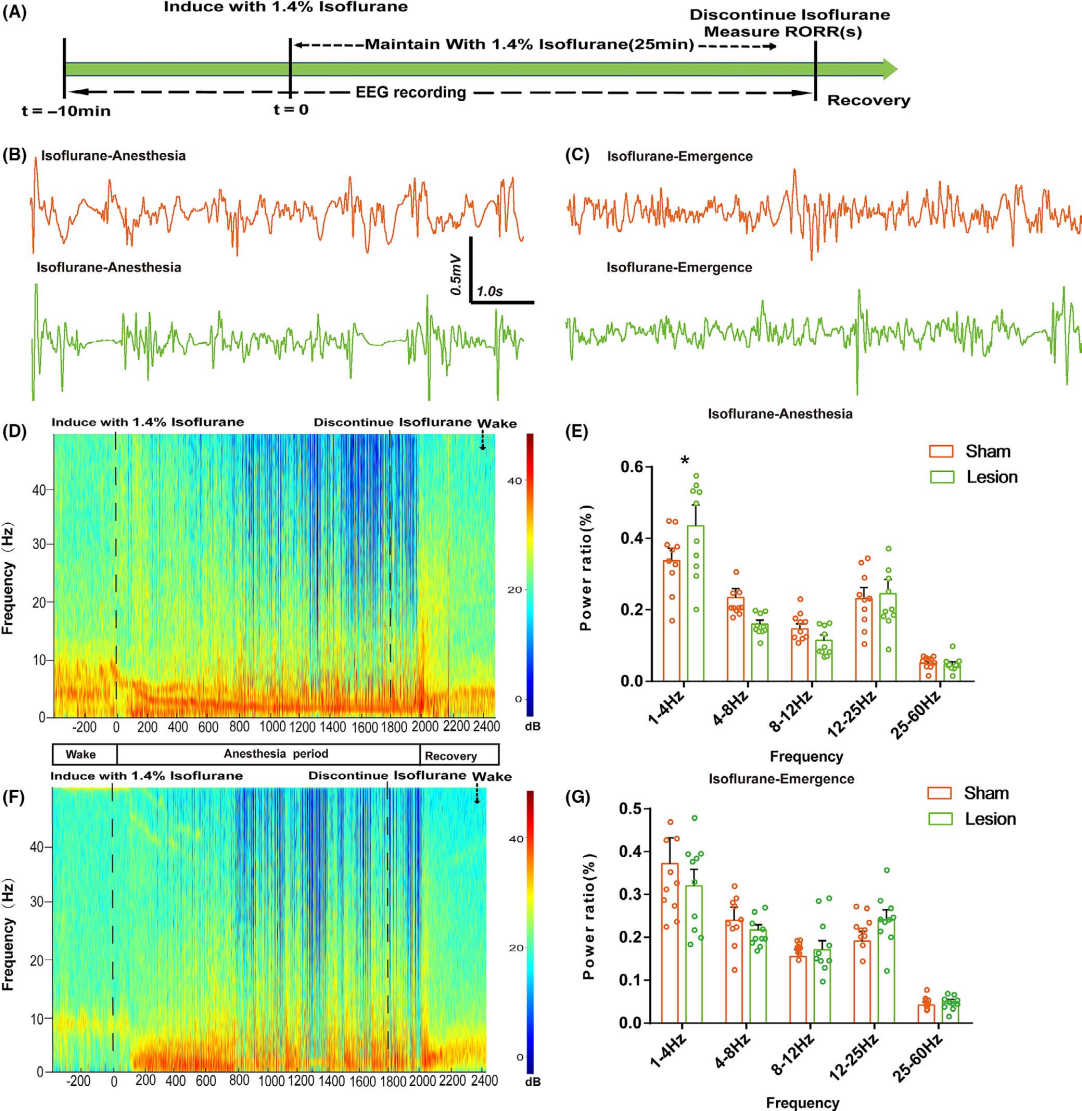
Lesion of DA neurons in vPAG affected the induction time and the emergence time companied with differential cortical spectral changes. A, Timeline for EEG recording in lesion and sham groups during isoflurane anesthesia. B, C, Representative electroencephalogram (EEG) activity waveforms in the two group. D, Spectrograms of EEG power during isoflurane anesthesia and emergence period in the lesion group. E, Power ratio of the δ band (1‐4 Hz) in the lesion group was higher than in the sham group during isoflurane anesthesia. F, Spectrograms of EEG power during isoflurane anesthesia and emergence period in the sham group. G, Lesion to DA neurons in the vPAG had no impact on the emergence period between the two groups (n = 10; mean ± SD; *P < .05 by independent t test)

As shown in Figure 2A, the DA neuron count in the vPAG was 66.2% lower after 6‐OHDA treatment than in the normal group (46.10 ± 7.84 mm^2^ vs. 136.40 ± 11.65 mm^2^, respectively; *P* < .0001; Figure 2C). Moreover, the induction time of isoflurane anesthesia noticeably accelerated (134.60 ± 21.27 seconds vs. 191.90 ± 31.65 seconds, respectively; *P* < .001; Figure 2D) and the recovery time was significantly slower (404.80 ± 77.89 seconds vs. 474.9 ± 65.01 seconds, respectively; *P* < .05; Figure 2E). Meanwhile, cortical EEG in the prefrontal cortex of the sham and lesion groups was recorded during isoflurane anesthesia. During isoflurane‐induced anesthesia, the power ratio of the δ wave was notably higher in the lesion group than in the sham group (0.435% ± 0.165% vs. 0.337% ± 0.982%; *P* = .032; Figure 3D,E), whereas the other bands did not differ significantly between the two groups. As illustrated in Figure 3F,G, there seems to be a downward trend on the power ratio of the δ wave in the lesion group, but lesion to the DA neurons in the vPAG had no statistical significance on the EEG spectral power ratio during the emergency period.

### 3.3 Activation/inhibition of GABA_A_ receptors in vPAG altered the induction and emergence time from isoflurane anesthesia

**Figure 4:**
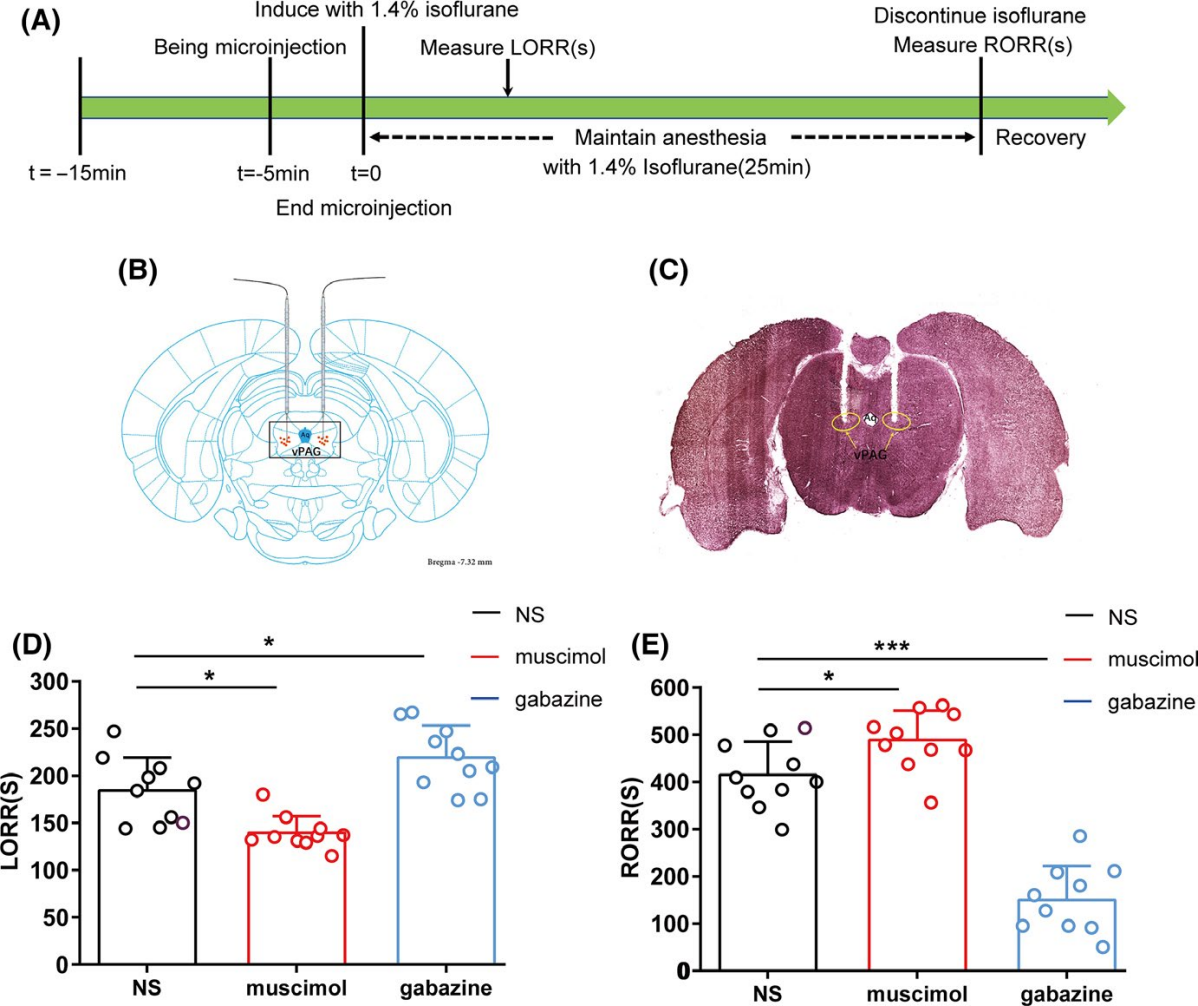
The effects of muscimol and gabazine in the vPAG on the LORR and RORR times of isoflurane anesthesia. A, Timelines of isoflurane anesthesia‐related behavioral tests measuring induction time (LORR) and emergence time (RORR). Rats received a microinjection of saline and GABA_A_R agonist/antagonist, respectively. B, Schematic of cannula sites confirmed from the rat brain atlas (Paxinos & Watson, 2007). The orange dots represent infusion sites within the vPAG. C, Representative slice showing the microinjection position (yellow circle) located in the vPAG. D, E, Microinjection of muscimol shortened the induction time and prolonged the emergence time of isoflurane anesthesia, compared to the control microinjection of saline. Microinjection of gabazine postponed the induction and significantly accelerated the emergence time of isoflurane anesthesia (*P < .05; **P < .01; ***P < .0001; n = 10 per group)

We investigated whether vPAG GABA_A_ receptors participate in the regulation of isoflurane anesthesia (n = 10 in each group). Figure 4A shows the experimental design to quantify LORR and RORR time for propofol anesthesia. The rat brains were removed to verify the injection sites using the rat brain atlas (Paxinos & Watson, 2007, Figure 4B,C). When the injecting sites were outside vPAG, the data were excluded. Compared with saline, microinjection of muscimol (GABA_A_R agonist) into the vPAG shortened LORR time (139.50 ± 17.67 seconds vs. 184.30 ± 35.10 seconds, respectively; *P* = .002; Figure 4E), while it prolonged RORR time (488.70 ± 62.56 seconds vs. 415.30 ± 69.73 seconds, respectively; *P* = .023; Figure 4D). Conversely, the LORR time from isoflurane anesthesia was prolonged by infusion of gabazine (GABA_A_R antagonist) into the vPAG (220.11 ± 36.15 seconds vs. 184.30 ± 35.10 seconds, respectively; *P* = .043; Figure 4E), and RORR time was significantly shortened (150.20 ± 71.77 seconds vs. 415.30 ± 69.73 seconds, respectively; *P* < .0001; Figure 4D). Based on the above results, we inferred that GABA_A_ receptors in the vPAG modulate isoflurane anesthesia.

### 3.4 GABA_A_ receptors of vPAG led to cortical spectral changes during isoflurane anesthesia

**Figure 5:**
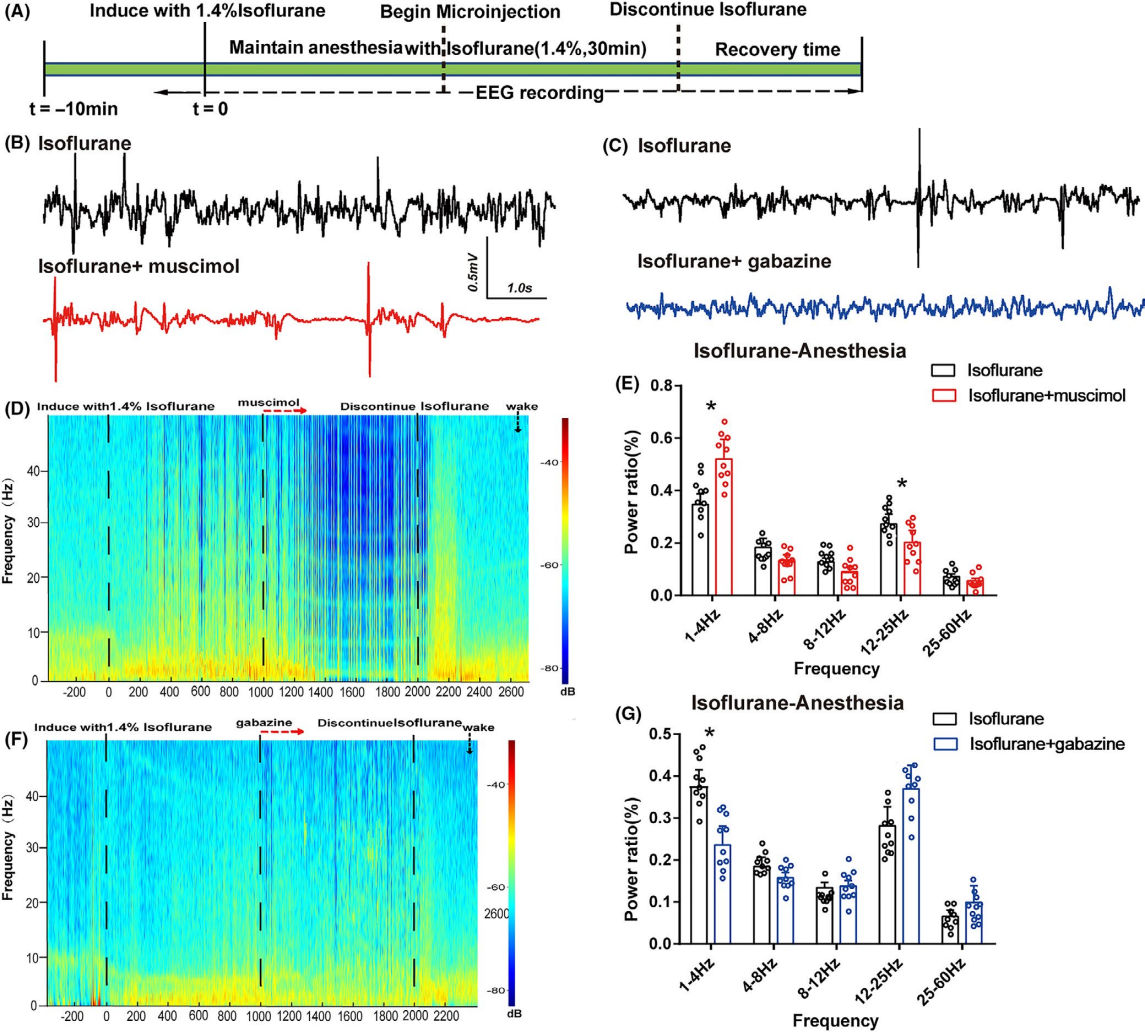
Power spectral analysis of cortical EEG before and after muscimol or gabazine during isoflurane anesthesia. (A) Timeline for EEG recording in microinjection experiments. (B)The representative example of EEG activity waveforms and corresponding EEG power recorded in the PFC before and after infusion of (B, D) muscimol or (C, F) gabazine during isoflurane anesthesia. (E) Activation of muscimol in the vPAG augmented δ band (1‐4 Hz) and suppressed the power ratio in the β band (12‐25Hz) during isoflurane anesthesia. (G) In contrast, blockade of GABA_A_R decreased the δ band during isoflurane anesthesia. Data from spectral analyses of EEG power are presented as mean ± SD (n = 10). *P < .05

The EEG recording was employed to further assess how the GABA_A_ receptors in the vPAG affect the processes of isoflurane anesthesia. Administration of muscimol into the vPAG augmented the power ratio of δ waves (1‐4 Hz) and declined the power ratio in β band (12‐25Hz) during isoflurane anesthesia (Figure 5E, *P < *.05), without affecting another bands (Figure 5B,D). Interestingly, gabazine treatment just reduced the power ratio of δ band and had no effect on either β band or other bands (Figure 5C,F,G). Consequently, vPAG activation via GABA_A_ receptors results in cortical arousal during isoflurane anesthesia.

### 3.5 GABA_A_R agonist/antagonist regulated isoflurane‐induced inhibition of vPAG‐DA neurons

**Figure 6:**
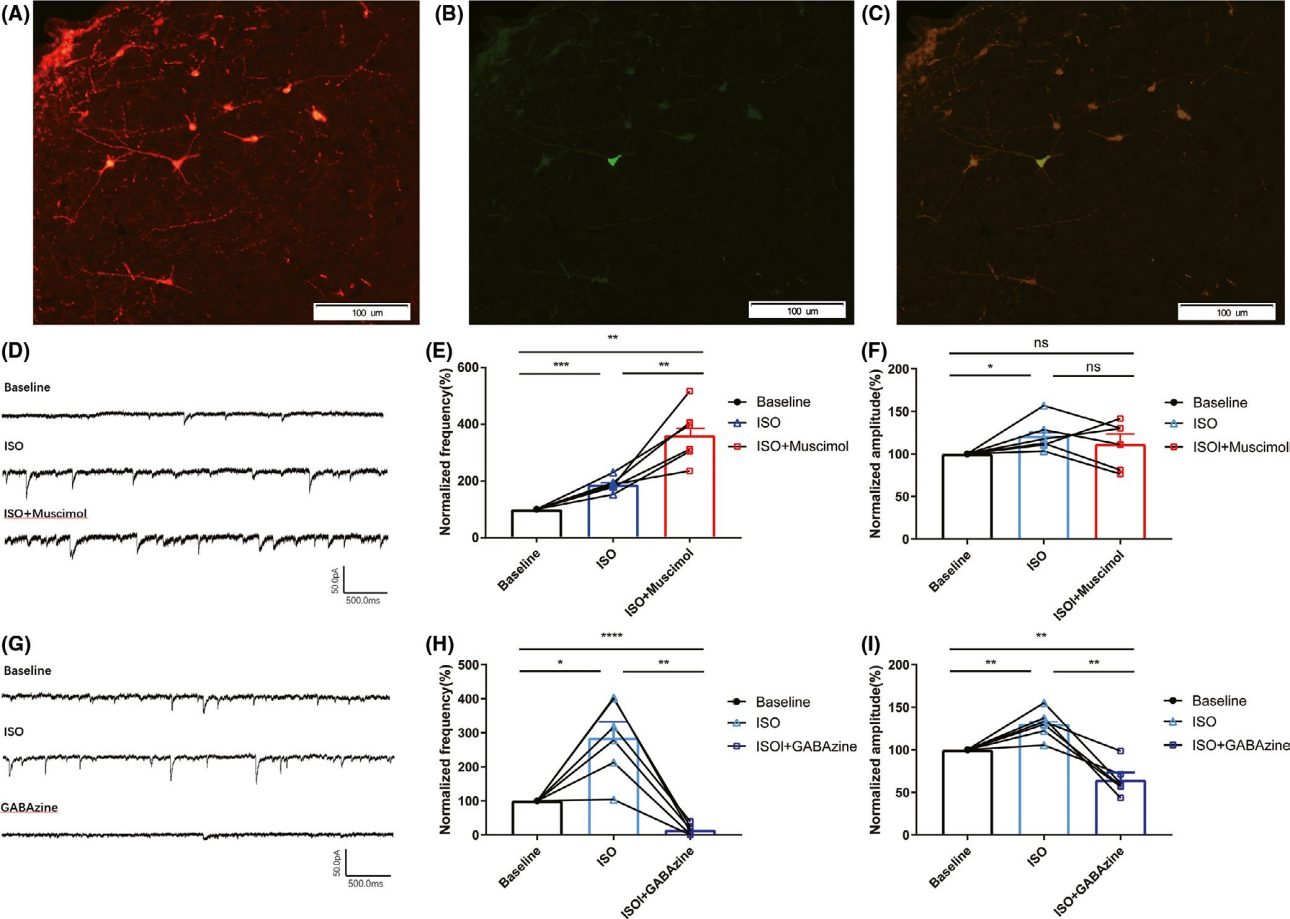
GABA_A_R affected isoflurane‐induced increases in GABAergic transmission. A, Immunofluorescent labeling of vPAG‐DA neurons: tyrosine hydroxylase‐positive neurons are stained in red. B, Lucifer yellow‐labeled neurons in the vPAG are shown in green after whole‐cell patch clamp recording under fluorescent microscopy. C, Lucifer yellow‐labeled neuron merged with tyrosine hydroxylase‐positive neurons. Scale bar: 100 µm. D, G, Sample current clamp traces showing spontaneous inhibitory postsynaptic currents (sIPSC) in vPAG‐DA neurons. E, Normalized frequency and F, amplitude of six neurons at baseline (black circles), during isoflurane exposure (blue triangles), and during concomitant isoflurane and muscimol exposure (red squares). H, Normalized frequency and I, amplitude of six neurons at baseline (black circles), during isoflurane exposure (blue triangles), and during concomitant isoflurane and gabazine exposure (dark blue squares). Connected points represent paired responses of individual neurons. All data are expressed as mean ± SD. Data were analyzed using one‐way ANOVA and post hoc Bonferroni‐corrected comparisons. (*P < .05; ** *P* < .01, *** *P* < .001, **** *P* < .0001; ns = not significant)

Whole‐cell voltage‐clamp recording was used to explore the effect of GABA_A_R agonist/antagonist on sIPSC, which reflects GABAergic transmission of vPAG‐DA neurons. As shown in Figure 6A‐C, we recorded 12 sets of vPAG‐DA neuron data from 83 neurons, and we also used AP5 and CNQX to block glutamate receptors. Infusion of 1.4% isoflurane prominently increased the sIPSC frequency by 285.78% ± 115.25% (*P* = .011; n = 6) and the amplitude by 130.54% ± 18.16% (*P* = .006; n = 6) in DA neurons, while gabazine significantly reduced the isoflurane‐induced augmentation of frequency (204.22% ± 59.96% vs. 14.47 ± 5.97%; *P* < .0001; Figure 6G,H) and the amplitude of sIPSC (133.29% ± 16.51% vs. 60.67 ± 14.00%, *P* = .003; n = 6; Figure 6I). Conversely, muscimol significantly enhanced the isoflurane‐induced augmentation of frequency (187.96% ± 25.28% vs. 361.94% ± 98.84%, *P* = .006; n = 6; Figure 6D,E), while the amplitude of sIPSC did not differ significantly between the isoflurane and isoflurane plus muscimol groups (Figure 6F; *P* > .05; n = 6). Based on these results, we concluded that isoflurane probably inhibits the neuronal activities of vPAG‐DA neurons by activating the GABA_A_ receptor and that GABA_A_R agonists/antagonists affected the facilitatory effects of isoflurane on GABAergic transmission primarily through presynaptic and postsynaptic mechanisms.

## 4 DISCUSSION

**Figure 7:**
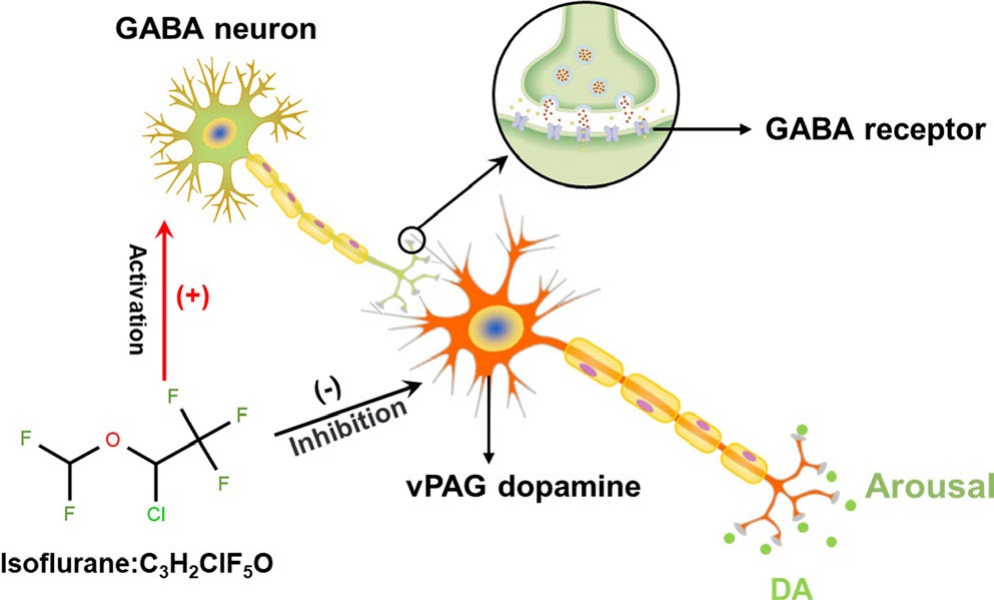
Proposed model for vPAG‐DA modulate isoflurane anesthesia in rats: Isoflurane activates GABA_A_ receptor to act on GABA neuron and vPAG‐DA neurons. Then, isoflurane directly suppressed the activity of wake‐promoting vPAG‐DA neurons to induce anesthesia or activate GABA neuron to indirectly inhibit vPAG‐DA neurons causing the reduction of DA

In the present study, we used behavioral testing, calcium fiber photometry recordings, and electrophysiology in vitro and in vivo to confirm that neural activity in the vPAG is altered during anesthetized states, that vPAG‐DA neurons are involved during induction and emergence from isoflurane anesthesia, and that isoflurane probably suppresses the activity of vPAG‐DA neurons by activating GABA_A_ receptor (as shown in Figure 7).

Using calcium fiber photometry recordings, we first found that vPAG neurons displayed an attenuated calcium signal in the transition from wakefulness to isoflurane‐induced LORR, implying that isoflurane‐induced anesthesia may require an inhibition of vPAG neurons to some degree. This idea is further supported by the finding that isoflurane significantly increased the release of presynaptic inhibitory neurotransmitter GABA in vPAG‐DA neurons via increasing the frequency and decay time of sIPSC. In the recovery period, vPAG neuronal activity was enhanced before arousal and that it was sharply activated in the transition from anesthesia to arousal, suggesting that vPAG neurons participated in the arousal, and was highly activated at the emergence moment. Similar results were observed a previous study that half of the vPAG‐DA neurons expressed c‐Fos in the natural wake but none in sleep.^15^ Additionally, Eban‐Rothschild et al used fiber photometry recordings to show that the activity of VTA dopaminergic neurons started to decline before the wakefulness to nonrapid eye movement (NREM) transition and that they augment their activity before NREM‐to‐arousal transitions via fiber photometry recordings.^6^ Recently, chemogenetic activation of locus coeruleus (LC) inputs to vPAG‐DA neurons, or of vPAG‐DA neurons directly, delayed sleep latency and promoted arousal in mice.^14^ These results also suggest that vPAG‐DA neurons have a significant influence on modulating isoflurane anesthesia.

To explore the role of vPAG‐DA neurons, 6‐OHDA was utilized to directly ablate the DA neurons, which resulted in an acceleration of isoflurane anesthesia induction and a slackening of reanimation. This was consistent with our previous study, which showed that unilateral ablation of vPAG‐DA neurons shortened the induction time and prolonged the recovery time in propofol anesthesia.^16^ Taken together, these results suggest that DA neurons in the vPAG play an equally critical role on both propofol and isoflurane anesthesia.

According to our previous works, the suppressing effect of propofol on vPAG‐DA neurons promotes presynaptic GABA release and enhances postsynaptic GABA_A_ receptor sensitivity. Similarly, the GABA_A_ receptor also involved in the isoflurane‐induced anesthesia^19^; hence, we investigated the role of GABA_A_ receptors in the vPAG to isoflurane anesthesia. Our study also found that intra‐vPAG injection of gabazine delayed induction and accelerated arousal, suggesting the involvement of GABAergic projections in modulating vPAG‐DA neurons. The notion is supported by that the vPAG has large source of inhibitory inputs from both the VTA and VLPO.^15^, ^20^, ^21^ It has been reported that VTA projections to the vPAG had an approaching 50% of GABAergic neurons, indicating that vPAG mainly received GABAergic projections from VTA in rats.^22^ Additionally, the ventrolateral preoptic area (VLPO), a mainly GABAergic regions to modulate sleep‐wake transitions, also has massive projections to vPAG‐DA neurons.^15^, ^20^ Recently, chemogenetic activation of GABA neurons in the VTA promoted NREM sleep with higher delta power in mice.^23^ In addition, selective ablation or suppression of GABAergic neurons in VMP where includes VTA and rostomedial tegmental nucleus increased wakefulness through disinhibiting dopaminergic systems, while special activation of VMP GABAergic neurons strongly increased SWS and drastically decreased wakefulness.^24^ We speculated that the vPAG‐DA neurons may be suppressed by inhibiting extrinsic GABAergic activity during isoflurane anesthesia, and microinjection of the GABA_A_R antagonist might disinhibit the inhibition of vPAG‐DA neurons, implying that GABA receptors involved in regulating the isoflurane‐induced anesthesia.

Meanwhile, intra‐vPAG injection of gabazine significantly decreased the emergence time from isoflurane anesthesia, accompanied by reduction of the δ wave, while muscimol decreased the EEG power in the β band and increased δ band. Ablation of DA neurons in vPAG only attenuated the power ratio of δ wave in isoflurane anesthesia maintenance period but not affect the emergence period, which in line with that photostimulation of VTA DA neurons specially decrease the δ band during the emergence period of isoflurane anesthesia.^13^ The unilateral lesion to vPAG‐DA neurons did not completely decrease vPAG‐DA neurons, which maybe lead to prolong the emergence time but have no statistical significance on the EEG during recovery period between the two groups. In one study, when glutamatergic neurons in PVT were optically excited under isoflurane anesthesia, δ wave were changed and the emergence was accelerated.^25^ Additionally, in another study, application of NE into the central medial thalamic (CMT) declined δ power in the frontal cortex of propofol‐anesthetized rats, without affecting the power in other frequency bands.^26^ These findings suggest that EEG δ wave closely related to anesthetic levels, and we found a direct role that the activation of vPAG‐DA neurons initiated the arousal of prefrontal cortex, as evidenced by a reduction of δ‐band power.

To further investigate the underlying mechanism, whole‐cell current patch clamp was used to record GABA_A_ receptors mediated sIPSC. Data showed that isoflurane (0.50 mmol/L) increased the frequency and amplitude of sIPSC, while administration of GABA_A_R antagonist sharply decreased isoflurane‐induced the augment of frequency and amplitude of sIPSC and administration of GABA_A_R agonist further increased the frequency of sIPSC. Generally, sIPSC, GABA_A_ receptor‐mediated Cl– currents, is produced by presynaptic GABA acting on postsynaptic GABA_A_ receptor. The frequency of sIPSC reflects the release of presynaptic transmitter, while the amplitude reflects postsynaptic reactivity elicited by the release of neurotransmitter from vesicles.^27^ Consistent with our findings, Nishikawa and colleagues^28^ found that isoflurane differentially enhanced the mean amplitude, frequency, and prolonged the decay time of sIPSC mediated by GABA_A_ receptor in rat hippocampal interneurons. Previous work has shown that isoflurane did alter spontaneous inhibitory GABAergic neurotransmission to parasympathetic cardiac vagal neurons (CVNs) at both postsynaptic and presynaptic sites through decreasing the frequency and increasing the decay time of sIPSC in CVNs.^29^ Therefore, our results suggested that isoflurane facilitates GABAergic transmission in vPAG‐DA neurons through a potentiation of GABA_A_ receptor‐mediated Cl^–^ currents on both presynaptic and postsynaptic sites, while that GABA_A_R antagonist evidently inhibited GABAergic transmission in vPAG dopamine neurons, which eventually disinhibits the activity of vPAG‐DA neurons. Conversely, GABA_A_R agonist enhanced isoflurane‐induced inhibition. Based on our results, we believe that hyperpolarization of vPAG‐DA neurons and increase of GABA release from the presynaptic and postsynaptic may be part of the mechanism that suppression of vPAG‐DA neurons delayed emergence from isoflurane anesthesia and elicits changes in the EEG.

The present study contains some limitations. Firstly, the vPAG area has a number of neuron populations. Although GABA_A_ receptors are present in the vPAG‐DA^15^, nonspecifically activating/inhibiting of vPAG neurons does not exactly address the function of DA neuron populations. Selective techniques for further analysis on the function of specific neuron populations are needed. Secondly, the effects of isoflurane on global oxidative metabolism and cerebral blood flow^30^ that accompany induction and emergence period may also affect behavioral results. Finally, the vPAG also sends brain‐wide projections, including direct projections to the cortex.^31^ Anesthesia decouples signaling along apical dendrites of layer 5 pyramidal neurons, indicating that anesthesia have strong impact on the brain connectivity.^32^ Additionally, different anesthetics may have differential effects on the consciousness and resting state of brain connectivity in animals.^30^ We need further clarify the role of neurons in different anesthetics and vPAG dopaminergic pathways involved in general anesthesia.

Taken together, we have provided multiple lines of evidence to support the idea that vPAG‐DA neurons have a functional role in modulating isoflurane anesthesia and the neuronal activities of vPAG‐DA neurons were suppressed by isoflurane via activating GABA_A_ receptor.

## CONFLICT OF INTEREST

The authors declare that there are no conflicts of interest in the authorship or publication of the contribution.

## ETHICS APPROVAL AND CONSENT TO PARTICIPATE

All experimental and surgical procedures were approved by Committees on Investigations Involving Animals in Zunyi Medical University, China (grant number: 2019(2)‐289).

## Data Availability

The datasets generated and analyzed during the current study are available from the corresponding author on reasonable request.
